# Comparative analysis among three Taiwan-specific *Gentiana* species and Chinese medicinal plant *Gentiana scabra*

**DOI:** 10.1186/1999-3110-54-54

**Published:** 2013-11-05

**Authors:** Shih-Hung Huang, Emily hin-Fun Chen, Chin-Tung Wu, Chao-Lin Kuo, Hsin-Sheng Tsay

**Affiliations:** 1 Department of Applied Chemistry, Chaoyang University of Technology, Taichung, Taiwan; 2 Department of Agronomy, National Chung Hsing University, Taichung, Taiwan; 3 Department of Computer Science and Information Engineering, Providence University, Taichung, Taiwan; 4 Department of Chinese Pharmaceutical Sciences and Chinese Medicine Resources, China Medical University, Taichung, Taiwan

**Keywords:** Antioxidant, *Gentiana davidii* var. *formosana*, *Gentiana scabra*, Gentiopicroside, Longdan

## Abstract

**Background:**

The root of *Gentiana scabra* is commonly known as Longdan in Chinese herbal medicines and has been used in the treatment of inflammation, anorexia, indigestion and gastric infections for over 2000 years. High market demand had made *G. scabra* (GS) plants not to be the only source of Longdan in China, other *Gentiana* spp., *G. triflora, G. manshurica* and *G. rigescens*, were also recognized as Longdan in China now.

**Results:**

In this study, we identified three Taiwan-specific *Gentiana* spp., *G. davidii var. formosana* (GDF) and *G. arisanensis* (GA) and *G. scabrida var. punctulata* (GSP) that are phylogenetically different from GS (main source of Longdan). However, the active compounds of Longdan, gentiopicroside and swertiamari, were found in GSP and GDF showed higher antioxidant ability and free radical scavenging activities than Chinese Longdan. This discovery might explore the medicinal potential of GDF. Meanwhile, another Taiwan-specific *Gentiana* spp., GSP, was found to have the strongest antioxidant ability and free radical scavenging activities which might suggest a possible use of GSP as a source of natural antioxidant agents for industrial purpose.

**Conclusions:**

The finding of this study indicated that ITS analysis can be used to identify Taiwan-specific *Gentiana* spp. Also the Taiwan-specific *Gentiana* spp. which has strongest antioxidant and free radical scavenging activities among others could be a better choice for industrial purpose.

**Electronic supplementary material:**

The online version of this article (doi:10.1186/1999-3110-54-54) contains supplementary material, which is available to authorized users.

## Background

The genus *Gentiana* is a large genus comprised about 400 species which are widely distributed in temperate regions of Asia, Europe, the Americas, northwest Africa, eastern Australia and New Zealand (Georgieva et al., 2005; Zając and Pindel, 2011). Gentian root is used in the production of wines, liqueurs, and bitter flavoring in Europe and Australia. In Asia, the root of *Gentiana scabra*, is commonly known as Longdan in Chinese herbal medicines and has been used in the treatment of inflammation, anorexia, indigestion and gastric infections (Tang and Eisenbrand, 1992) for over 2000 years. Recent studies indicated that the root extract from *Gentian* plant can inhibit tumor cell proliferation (Matsukawa et al. 2006), enhance DNA repair, exerts antioxidant activity (Hudecová et al., 2012) and hepatoprotective effect (Ko et al., 2011).

While the market demands for *G. scabra* (GS) plants have greatly increased over the past decades, their supplies are now in severe shortage due to the over-exploitation and ecological destruction of their natural habitats. Now the roots of *G. scabra* (GS) is not the only source of Longdan in China, but also the roots from other *Gentiana* spp., *G. triflora, G. manshurica* and *G. rigescens*. In Taiwan, there are more than 10 *Gentiana* spp. Most of them are annual herbs, except *G. davidii var. formosana* (GDF) and *G. arisanensis* (GA). Moreover, there is one species with tall phenotype, *G. scabrida* var. *punctulata* (GSP) which is sometimes up to 20 cm while others are less than 15 cm tall (Chen and Wang, 1999). Hence, we took GDF, GA and GSP as our research material in order to explorer their medicinal potential.

Chemical investigation of root extract of *Gentiana* spp. has resulted in isolation of a series of loganic acid, swertiamarin, gentiopicroside, gentisin and isogentisin (Aberham et al., 2007; Aberham et al., 2011). Gentiopicroside (a secoiridoid glucoside) and swertiamarin are two important active components used for gentian identification.

Another method to identify or differentiate *Gentiana* spp. is rDNA ITS (internal transcribed spacers) sequence analysis (Ji et al., 2003). The ITS region in rDNAs comprises of ITS1 and ITS2. ITS1 is between the 18S and 5.8S rDNA, while ITS2 is between the 5.8S and 28S rDNA. The conserved regions of 18S and 28S rDNA have been used to design universal primers used to amplify the flanking ITS regions (Wu et al., 2012). Since ITS1 and ITS2 regions can be amplified by using universal primers, and the results are reliable, rDNA ITS sequence analysis was used in our study to understand the phylogenetic relationship of selected Taiwan-specific *Gentiana* spp. (GDF, GA and GSP) with Chinese Longdan.

Based on the recent studies about the root extract from Longdan in enhancing DNA repair and exerting antioxidant activity, we were also interested in the possible antioxidants such as polyphenols and flavonoids existed in three Taiwan-specific *Gentiana* spp. (GDF, GA and GSP). Free radicals and reactive oxygen species (ROS) such as superoxide, hydroxyl and peroxyl radicals produced during oxidation (Blokhina et al., 2003) or by exposure to radiation, toxic chemicals, smoking, alcohol and oxidized polyunsaturated fatty acids have been implicated to cause protein, DNA and cell membranes (Farber, 1994) and leading to the development of a variety of diseases such as cardiovascular disease, cancer and other chronic diseases (Willcox et al., 2004). Thus, measurement of total phenolic and flavonoids, as well as antioxidant and free radical scavenging activity have become important tools to study the differences in our three Taiwan-specific *Gentiana* spp. (GDF, GA and GSP) and Chinese Longdan (GS).

In the present study, the identification of three Taiwan-specific *Gentiana* spp. (GDF, GA and GSP) based on rDNA ITS sequence analysis and HPLC method was performed. Meanwhile, the ethanol extracts of the roots from three Taiwan-specific *Gentiana* spp. (GDF, GA and GSP), Chinese Longdan (GS) and ethanol extracts of two market-purchased dried gentian roots (herbal imported from China) were used to characterize their antioxidative potencies, scavenging activities against ABTS and DPPH radicals. The finding of this work may explore the medicinal and industrial potential of Taiwan-specific *Gentiana* spp.

## Methods

### Chemicals

1,1-Diphenyl-2-picrylhydrazyl (DPPH), phenazine methosulfate (PMS), sodium carbonate, gallic acid (GA), quercetin (QE), 2, 2-azinobis [3-ethylbenzothiazoline- 6-sulfonate] (ABTS), Folin-Ciocalteu’s reagent, were purchased from Sigma-Aldrich (St Louis, MO, USA). Swertiamarin and gentiopicroside were purchased from National Institute for Control of Pharmaceutical and Biological Products (Beijing, PR China). All other chemical reagents used were of analytical grade. The DNeasy Plant Mini kit was purchased from Qiagen (Hilden, Germany). The Plasmid DNA purification kit, DNA purification kit and DNA marker were purshased from GeneMark Technology (Tainan, Taiwan). Primers were synthesized by Tri-ibiotech (Taipei, Taiwan). The Fast-Run Taq Master Mix kit was purchased from Protech Technology Enterprise (Taipei, Taiwan).

### Plant material

**Figure 1:**
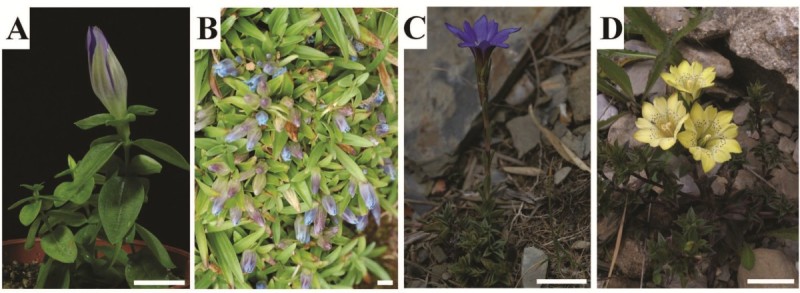
**Representative photographs of** ***Gentiana*** **spp. A**. *G. scabra* Bunge; **B**. *G. davidii* var. *formosana*; **C**. *G. arisanensis* Hayata; **D**. *G. scabrida* Hayata var. *punctulata*. Bar = 2 cm.

The *G. scabra* (GS) collected from China were grown in the green house of Chaoyang University of Technology (Taichung, Taiwan). *G. davidii* var. *formosana* (GDF), *G. arisanensis* (GA) and *G. scabrida* var. *punctulata* (GSP) were collected from Taichung, Nantou, and Jiayi counties in Taiwan (Figure 1). All plant materials were identified by Dr. Chao-Lin Kuo (associate professor and chairman of the Department of Chinese Pharmaceutical Sciences and Chinese Medicine Resources, China Medical University, Taichung, Taiwan). The dried gentian roots (DR1 and DR2) imported from China were purchased from a local medicinal plant market.

### DNA extraction, PCR amplification and sequencing

Genomic DNA was extracted from fresh leaves or dried roots. Approximately 100 mg of samples was pulverized with liquid nitrogen in a mortar and then extracted by DNeasy® Plant Mini Kit (Qiagen, Germany). The ITS1-5.8S-ITS2 regions of tested samples were amplified with the primer pair 18S-F (5′-CGT AAC AAG GTT TCC GTA GGT GA-3′) and 28S-R (5′-CCT TTC ATC TTT CCC TCG CGG T-3′) (Lin et al., 2007). PCR mixture containing 50 ng of genomic DNA, 1 μl of 18S-F primer, 1 μl of 28S-R primer and 25 μl of Taq Master Mix buffer was made up to 50 μl with sterile distilled water. The PCR programme consisted of an initial denaturising step of 5 min at 94°C followed by 35 cycles of 30 s at 94°C, 30 s at 55°C and 30 s at 72°C and a final extension step of 10 min at 72°C. Approximately 10 μl of PCR products were electrophoresed on 1% agarose gel, stained with ethidium bromide, and visualized under UV. The amplified PCR product was purified using illustra GFX PCR DNA and Gel Band Purification Kit (GE Healthcare Life Sciences) and subsequently sent for sequencing. All sequences obtained were characterized by using nucleotide blast (http://www.ncbi.nlm.nih.gov).

### Phylogenetic analysis

A total of 7 sequences of the ITS1-5.8S-ITS2 region of different *Gentiana* species collected from the NCBI databases, together with 3 additional ITS of GDF, GA and GSP obtained in this study were used for phylogenetic analysis. Phylogenetic analysis was performed by using the PHYLIP Version 3.69 (Felsenstein, 1989). Bootstrap analysis with 1000 times replicates (Felsenstein, 1985) of the alignment was applied to maximun parsimony method.

### Preparation of root extracts from *Gentiana* spp

All six *Gentiana* spp. roots were cut into small pieces, freeze-dried (24–48 h), and ground with a blender into fine powder form. Accurately weighed into 5.0 g aliquots and placed in 15 ml centrifuge tubes. After adding ethanol, the samples were sonicated at 40°C for 30 min and followed by centrifugation. Each sample was extracted three times and all the supernatants were collected and filtrated. Every filtrate was dried and resuspended in ethanol to the final concentration of 10 g/l.

### Determination of swertiamarin and gentiopicroside content

The HPLC system (Hitachi) equipped with L-2130 binary pump, an L-2200 auto-sampler and an L-2450 PDA-UV detector was used for the determination of swertiamarin and gentiopicroside. Swertiamarin and gentiopicroside were carried out by following the method of (Zhang et al., 2010) with some modifications. The chromatographic separation of analytes was performed at room temperature using a Mightysil RP-18 GP column (250 × 4.6 mm 5 μm). The mobile phase consisted of 0.2% phosphoric acid in water (solvent A) and 100% methanol (solvent B) flow rate of 1 ml/min. In the preliminary experiments, the elution conditions applied are as follows: 0–25 min, linear gradient 80-65% A; and, finally, reconditioning steps of the column was 80% A isocratic for 10 min. Data were collected and analyzed using EZchrom Elite Version 3.13 software. Various concentrations (1–100 mg/l) made from the standard stock of swertiamarin and gentiopicroside were used for calibration curve.

### Determination of total phenolic content

The amount of total phenolic was determined by the Folin-Ciocalteu method reported (Slinkard and Singleton, 1977) with slight modifications. Each extract (100 mg/l) was mixed with 200 μl distilled water and 40 μl of Folin-Ciocalteu phenol reagent, incubated at room temperature for 5 min and then mixed with 40 μl of 20% Na_2_CO_3_. After color development, the absorbance of all samples was measured at 765 nm using the UV–vis spectrophotometer. Gallic acid was used as standard and total phenolic content were expressed as mg/g gallic acid equivalent (GAE).

### Determination of total flavonoids

Total flavonoids were determined by AlCl_3_ method (Lamaison and Carnet, 1990). 100 μl of 2% AlCl_3_ was added to 100 μl of extract (100 mg/l). The mixture was vigorously shaken and followed by absorbance measurement at 430 nm. Quercetin was used as standard and total flavonoids were expressed as mg/g quercetin equivalent (QE).

### Determination of antioxidant activity

The antioxidant activity was determined by ABTS (Re et al., 1999), DPPH radical scavenging assay (Blois, 1958) and reducing power assay (Oyaizu, 1986).

DPPH radical-scavenging activity was determined as described with slight modification. Extracts in different concentration (0 to 1 g/l) were mixed with 100 mM Tris–HCl buffer (80 μl, pH 7.4), and 100 μl of the DPPH solution. The mixture was shaken vigorously and incubated for 30 min in the dark at room temperature. The absorbance was measured at 517 nm in a UV/Vis spectrophotometer. Ascorbic acid was used as a positive control. DPPH free radical scavenging ability (%) was calculated by using the following formula: Inhibition % = (1 – A/A_0_) × 100 where A_0_ is the absorbance at 517 nm of negative control, and A is the absorbance of mixture containing DPPH and sample.

The ABTS · ^+^ was produced by 7 mM ABTS stock solution with 2.45 mM potassium persulfate in water, which was kept in the dark at room temperature for 16 h to give the complete oxidation of ABTS. Before using, the ABTS · ^+^ solution was diluted with water to get an absorbance of 0.700 ± 0.050 at 734 nm. Briefly, 1 ml of ABTS · ^+^ solution was added to 30 μl of extract samples (10 to 200 mg/l) and mixed thoroughly. The reaction mixture was incubated at room temperature for 6 min and the absorbance was immediately recorded at 734 nm. The calibration curve was prepared by trolox solutions.

The Fe^3+^ reducing power of the extract was determined by the method described by Oyaizu (1986) with a slight modification. Different concentrations (10 to 200 mg/l) of the extract (0.5 ml) were mixed with 0.5 ml phosphate buffer (0.2 M, pH 6.6) and 0.5 ml potassium ferricyanide (0.1%), followed by incubation at 50°C for 20 min. After incubation, 0.5 ml of TCA (10%) was added to terminate the reaction. The upper portion of the solution (1 ml) was mixed with 1 ml distilled water, and 0.2 ml FeCl_3_ solution (0.1%). The reaction mixture was left for 10 min at room temperature and the absorbance was measured at 700 nm against an appropriate blank solution.

## Results

### Molecular identification and phylogenetic analysis

**Figure 2:**
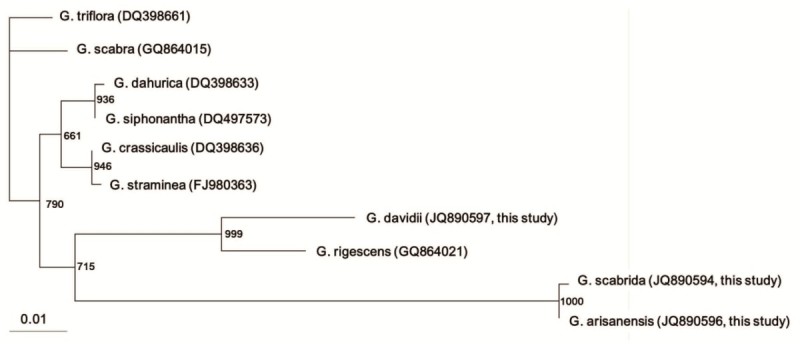
**Phylogenetic analysis of six different** ***Gentiana*** **spp.** Phylogenetic tree based on the sequences of ITS1-5.8S-ITS2 regions from different *Gentiana* spp. were constructed by Maximun Parsimony method (1000 bootstrap replicates).

A total of 7 sequences of the ITS1-5.8S-ITS2 region of different *Gentiana* spp. (GQ864021, DQ398633, DQ497573, DQ398636, DQ398661, FJ980363 and JQ890595) collected from the NCBI databases, together with 3 additional ITS of GDF (JQ890597), GA (JQ890596) and GSP (JQ890594) obtained in this study were used for phylogenetic analysis. All ITS were found to be 620–625 bp in length. A BLAST search of the ITS obtained in this study was similar to the ITS of GS (91-94% similarity, data not shown). However, the phylogenetic analysis based on ITS showed that they belong to different clusters. Three Taiwan-specific *Gentiana* spp. were phylogenetically different from two major Chinese Longdan (*G. scabra*, GS and *G. triflora*) (Figure 2) and close related to another Chinese Longdan (*G. rigescens*).

### Extraction yields

The ethanol extract yields from six different samples from *Gentiana* spp. (6.76%, 5.71%, 9.10%, 7.96%, 24.25% and 28.05% for GS, GDF, GA, GSP, dried root 1 (DR1) and dried root 2 (DR2) respectively) were shown in Table 1. Dried roots (DR1 and DR2) which are imported Chinese herbal showed higher extract yields based on the dry weight.

**Table 1:** **Ethanol extract yields from roots of different** ***Gentiana*** **spp.**

Samples	Extract yield (%)^a^
*G. scabra* (GS)	6.76
*G. davidii* var. *formosana* (GDF)	5.71
*G. arisanensis* (GA)	9.10
*G. scabrida* var. *punctulata* (GSP)	7.96
*Gentian* dried root 1 (DR1)	24.25
*Gentian* dried root 2 (DR2)	28.05

^a^Dry weight basis.

### Swertiamarin and gentiopicroside content

The calibration curves of swertiamarin and gentiopicroside showed good linearity (correlation coefficient = 0.9999 respectively) range from 0.1–100 mg/l (data not shown). The swertiamarin content in GDA (2.66 mg/g) and GS (2.52 mg/g) was the highest, followed by DR1 (1.06 mg/g). Others (GA, GSP and DR2) showed no swertiamarin content. On the other hand, GDF had the highest gentiopicroside (49.42 mg/g) content while comparing with DR1 (41.17 mg/g), GS (30.25 mg/g) and DR2 (9.13 mg/g). There is no gentiopicroside detected in GA and GSP (Table 2).

**Table 2:** **Swertiamarin and gentiopicroside contents in different** ***Gentiana*** **spp.**

Samples	Content (mg/g)^a^	
	Swertiamarin	Gentiopicroside
*G. scabra* (GS)	2.52 ± 0.03 A	30.25 ± 0.12 C
*G. davidii* var. *formosana* (GDF)	2.66 ± 0.12 A	49.42 ± 2.17 A
*G. arisanensis* (GA)	N. D.	N. D.
*G. scabrida* var. *punctulata* (GSP)	N. D.	N. D.
*Gentian* dried root 1 (DR1)	1.06 ± 0.03 B	41.17 ± 1.62 B
*Gentian* dried root 2 (DR2)	N. D.	9.13 ± 0.1 D

^a^Value are means ± standard error, n = 3. Means followed by the same letter are not significantly different at 5% level by LSD (least significant difference) test.

N.D.: Not detectable.

### Total phenolic content

The total phenolic contents of six sample extracts were determined and are presented in Table 3. The phenolic contents were calculated using mg GAE/g dry weight. Significant differences in total phenolic contents were observed in six samples. It ranged from 42.28 to 102.24 mg dry weight. Highest total phenolic content was observed in GSP followed by GDF. GS, GA and DR1 had similar and less content which were less than GSP.

**Table 3:** **Total phenolic and total flavonoids contents from ethanol extract of different** ***Gentiana*** **spp.** ^**a**^

Samples	Total phenolic^b^	Total flavonoids^c^
*G. scabra* (GS)	52.34 ± 0.73 CD	18.58 ± 1.30 B
*G. davidii* var. *formosana* (GDF)	66.31 ± 2.61 B	8.06 ± 0.52 C
*G. arisanensis* (GA)	42.28 ± 0.39 D	9.69 ± 0.80 C
*G. scabrida* var. *punctulata* (GSP)	102.24 ± 6.18 A	71.14 ± 4.16 A
*Gentian* dried root 1 (DR1)	43.17 ± 1.37 D	2.66 ± 0.10 D
*Gentian* dried root 2 (DR2)	60.59 ± 1.98 B	1.45 ± 0.14 D

^a^Value are means ± standard error, n = 3. Means followed by the same letter are not significantly different at 5% level by LSD (least significant difference) test.

^b^Expressed as mg gallic acid equivalent/g dry weight.

^c^Expressed as mg quercetin equivalent/g dry weight.

### Total flavonoid content

Flavonoid content of six sample extracts was determined by colorimetric method. Total flavonoid content (expressed as mg QE/g dry weight) ranged from 1.45 to 71.14 mg dry weight (Table 3). Highest total flavonoid content was observed in GSP followed by GS. DR1and DR2 had very low flavonoid content.

### Total antioxidant activity

**Figure 3:**
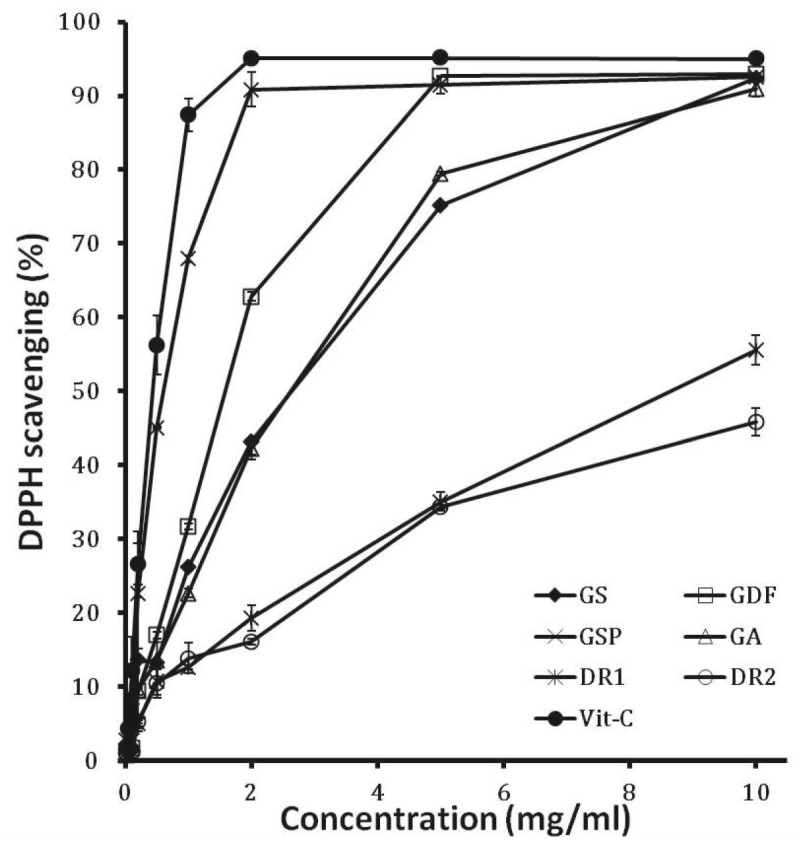
**DPPH radical scavenging activity (%) of ethanol extract from different** ***Gentiana*** **spp.** DPPH scavenging activity was tested with different concentrations of ethanol extracts from *Gentiana* spp. The results represent means ± standard deviation (n = 3).

Free radical scavenging activities of six sample extracts were assessed by the DPPH assay. A significant decrease in the concentration of DPPH radical was observed in Figure 3. The results showed that GSP had the highest DPPH scavenging activity with an IC50 value of 51.41 mg/l. IC50 values of GDF, GA and GS were 106.36, 182.24 and 201.07 mg/l respectively. Free radical removal capacity for DR1 and DR2 was weak.

**Figure 4:**
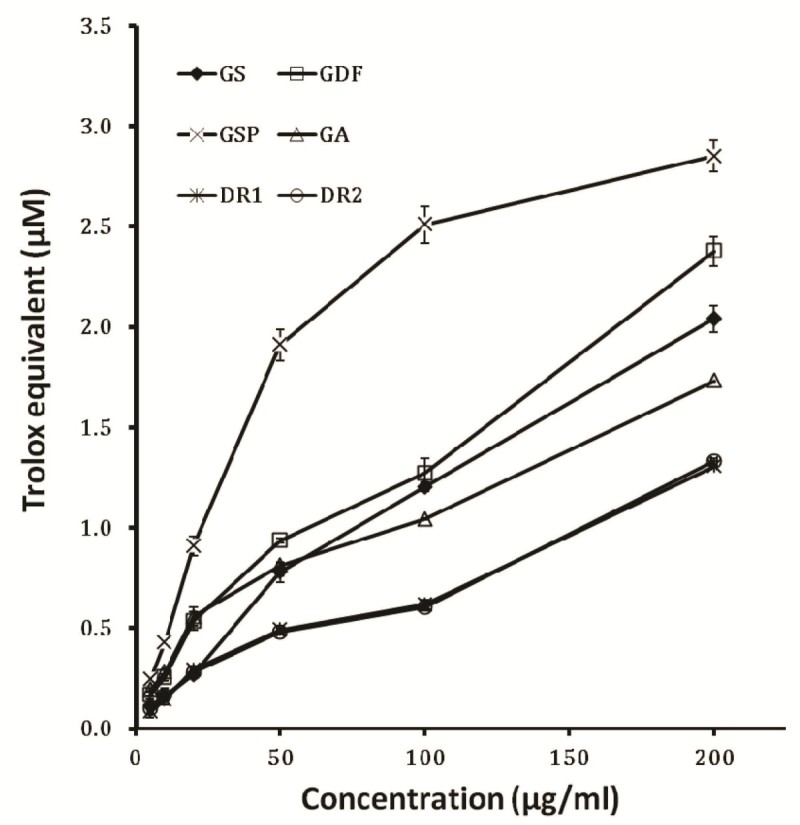
**Trolox equivalent antioxidant capacity (TEAC) of ethanol extract from different** ***Gentiana*** **spp.** TEAC carried out with different concentrations of ethanol extracts from *Gentiana* spp. The results represent means ± standard deviation (n = 3).

Total antioxidant activity of the six sample extracts increased with higher concentration of the extracts. Significant change was observed at 10 to 200 mg/l concentration of the extract (Figure 4). The total antioxidant activity of 200 mg/l GSP, GDF, GS, GA, DR2 and DR1 sample were 2.85, 2.38, 2.04, 1.73, 1.33 and 1.31 μM Trolox Equivalent respectively.

**Figure 5:**
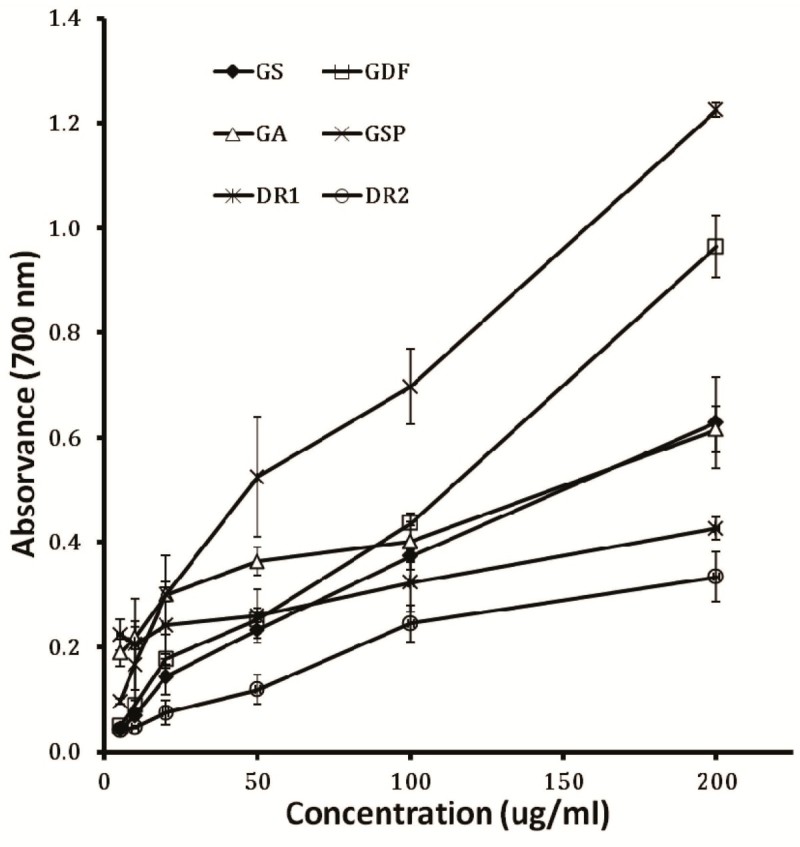
**Reducing power of ethanol extract from different** ***Gentiana*** **spp.** Reducing power was measured in different concentrations of ethanol extracts from *Gentiana* spp. The results represent means ± standard deviation (n = 3).

All six samples had shown a considerable amount of reducing activity. The reducing power increased with the higher concentration of gentian extracts and a significant change was observed at 10 to 200 mg/l concentration (Figure 5). 200 mg/l of the extracts showed absorbance values of 0.34, 0.43, 0.58, 0.62, 0.97 and 1.93 corresponding to DR2, DR1, GS, GA, GDF and GSP were respectively.

## Discussion

Plants belonging to Gentianaceae are used in wine production or as traditional medicines in many countries. Among them, the roots of *G. scabra* (GS), *G. triflora*, *G. manshurica* and *G. rigescens* are known as Longdan in Chinese herbal medicines. While the market demands for Longdan have greatly increased over the past decades, we tried to find the medicinal potential of three Taiwan-specific *Gentiana* spp.

Based on the ITS of three Taiwan-specific *Gentiana* spp. (GDF, GA and GSP) obtained in our study, previously published ITS of Longdan (*G. scabra* (GS), *G. triflora*, and *G. rigescens*) and of other *Gentiana* spp. (*G. dahurica, G. siphonantha, G. crassicaulis*, and *G. straminea*), we found that all of them showed high similarity to each other. However, phylogenetic analysis based on ITS showed that they belong to different clusters. We observed that GDF, GA and GSP are phylogenetically different from GS (main source of Longdan), hence we said they are Taiwan-specific *Gentiana* spp.

Based on the results obtained from determination of two active compounds (gentiopicroside and swertiamarin) existed in Longdan, we found 1.5 times more gentiopicroside in GDF than in GS. Moreover, the swertiamarin content in GDF and GS was similar, therefore Taiwan-specific spp. GDF may have medicinal effects potential as Chinese Longdan. Interestingly, DR1 and DR2 (Dried Longdan imported from China) were found with less active compounds than GDF. There is almost no gentiopicroside and swertiamarin in DR2. Probably, DR2 was not the authentic Longdan. Source of dried Chinese herbal is critical in some cases.

Since GDF might have medicinal potential, we tested its antioxidant capacity. Surprisingly GDF showed better antioxidant activity than Chinese Longdan, GS. This result might be correlated to its total phenolic content (66.31 mg GAE/g dry weight). Therefore we tested the DPPH radical scavenging ability and reducing power of GDF, and the results were as good as its antioxidant ability.

GSP (with tall phenotype as described previously (Chen and Wang, 1999) was detected with no gentiopicroside and swertiamarin. This result made it not to be a candidate of Chinese Longdan, however GSP had the strongest antioxidant ability, DPPH radical scavenging ability and reducing power than any others. The main reason for this should be its highest total phenolic (102.24 mg GAE/g dry weight) and flavonoids content (71.14 mg QE/g dry weight) which are 2 times and 4 times higher than Chinese Longdan (GS).

Phenolic compounds are the major constituents in most plants and was reported to possess antioxidant and free radical scavenging activities (Larson, 1988; Olajuyigbe and Afolayan, 2011). And free radicals are correlated to human diseases (Halliwell and Gutteridge, 1990).

Unfortunately, the high total phenolic content in DR1 and DR2 (Dried Longdan imported from China) did not enhance their antioxidant ability and reducing power. The possible explanation is the dryness or antibacterial treatment of Chinese herbal had affected our experimental results.

## Conclusion

The results from this study indicate that ITS analysis can be used to identify Taiwan-specific *Gentiana* spp. And one of the Taiwan-specific *Gentiana* spp., GDF, was found to posses Longdan specific active compound, and showed higher antioxidant ability and free radical scavenging activities than Chinese Longdan. This data might support the medicinal potential of GDF. Meanwhile, another Taiwan-specific *Gentiana* spp., GSP, was found to have the strongest antioxidant ability and free radical scavenging activities which might suggest a possible use of GSP as a source of natural antioxidant agents for industrial purpose.

## Authors’ original submitted files for images

Below are the links to the authors’ original submitted files for images. Authors’ original file for figure 1 Authors’ original file for figure 2 Authors’ original file for figure 3 Authors’ original file for figure 4 Authors’ original file for figure 5
