# Supplementary figures and images for: Comparative analysis among three Taiwan-specific Gentiana species and Chinese medicinal plant Gentiana scabra
Source: Bot Stud. 2013 Nov 5;54:54. doi: 10.1186/1999-3110-54-54 (PMC5430382; doi:10.1186/1999-3110-54-54)

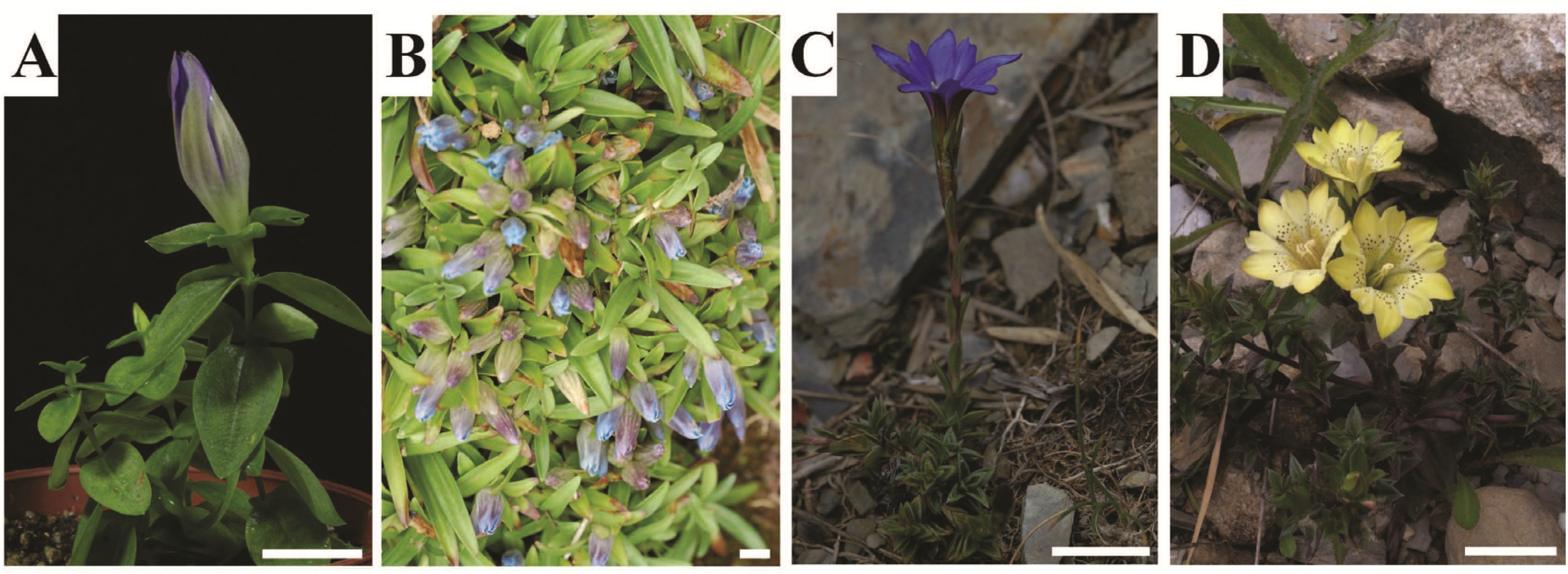

Supplement: Supplementary file 1 — Authors’ original file for figure 1 [file 40529_2013_46_MOESM1_ESM.jpeg]

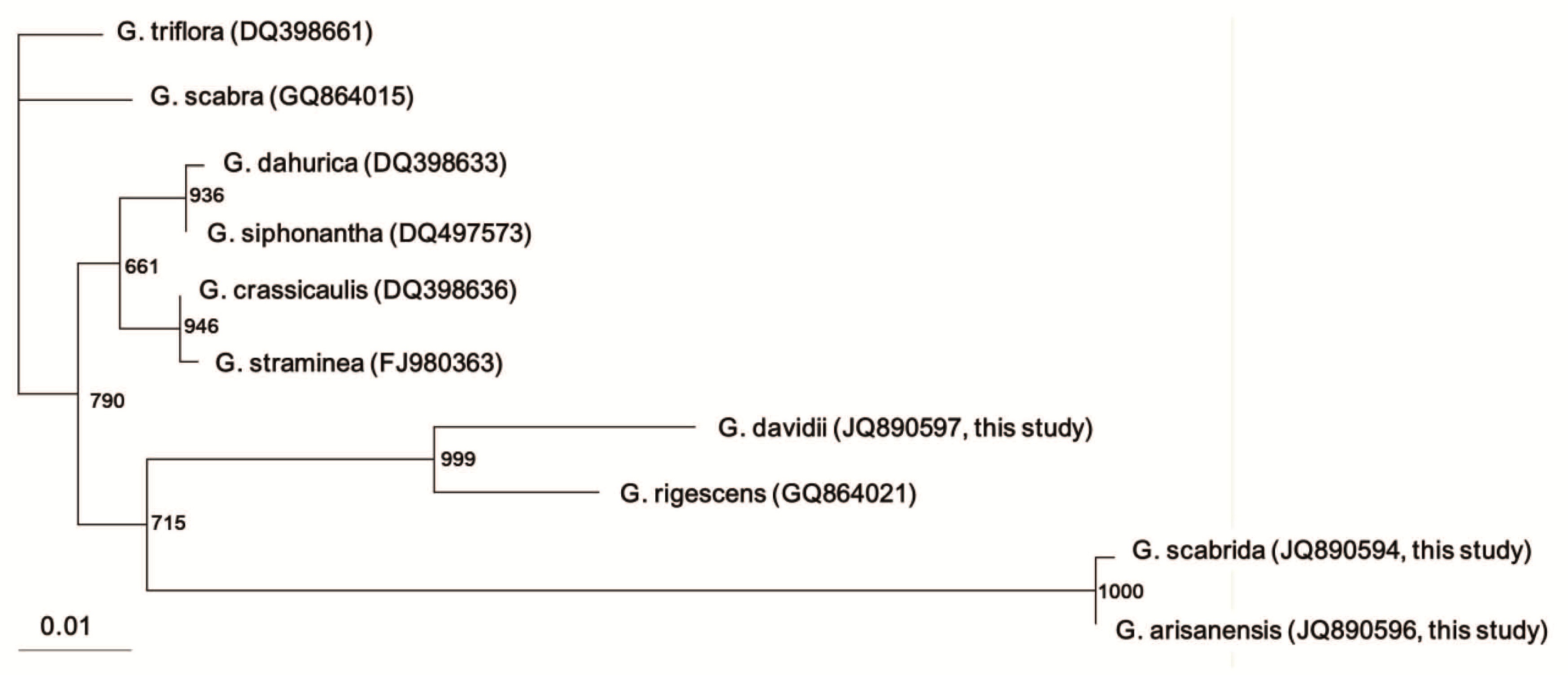

Supplement: Supplementary file 2 — Authors’ original file for figure 2 [file 40529_2013_46_MOESM2_ESM.jpeg]

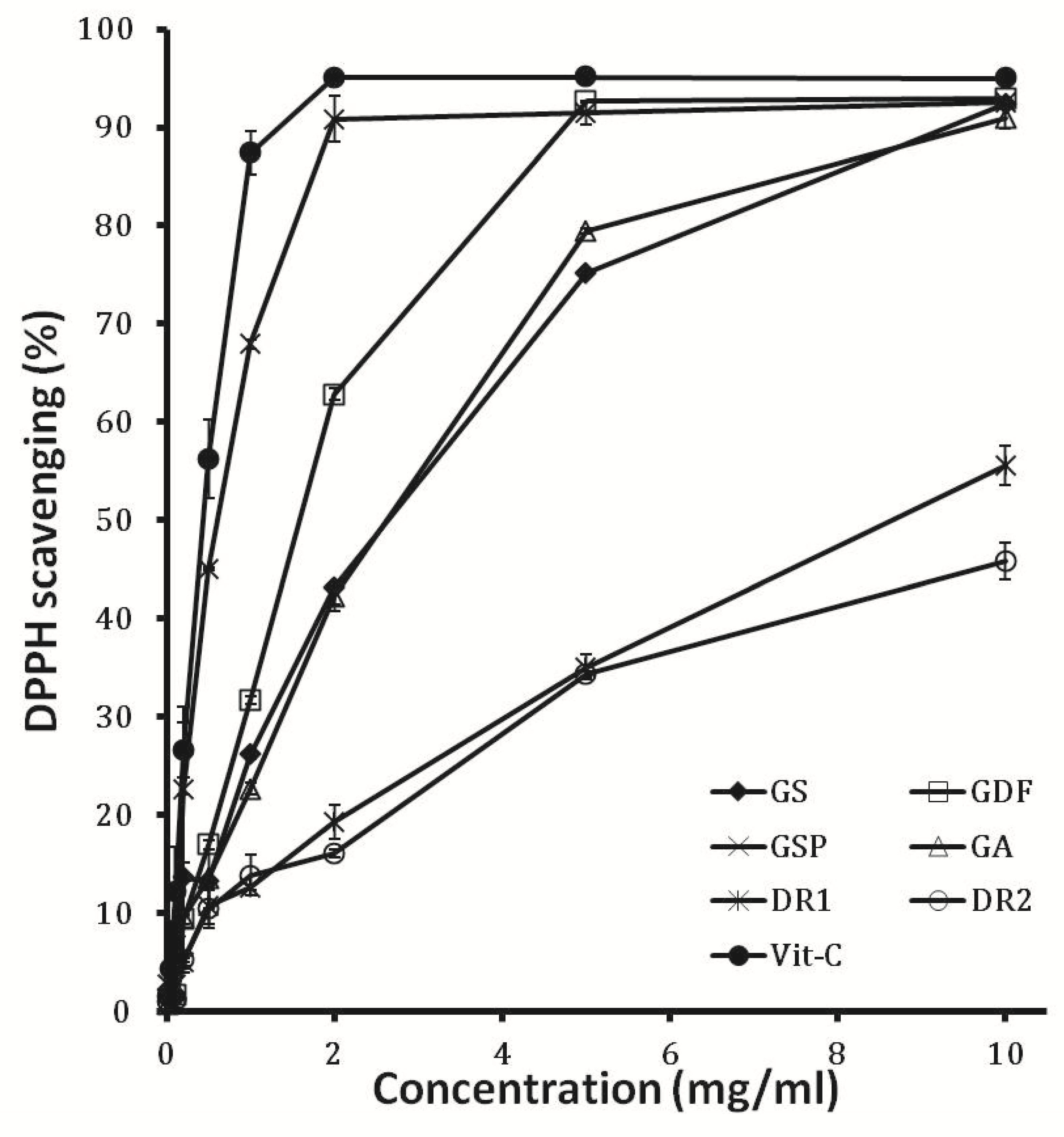

Supplement: Supplementary file 3 — Authors’ original file for figure 3 [file 40529_2013_46_MOESM3_ESM.jpeg]

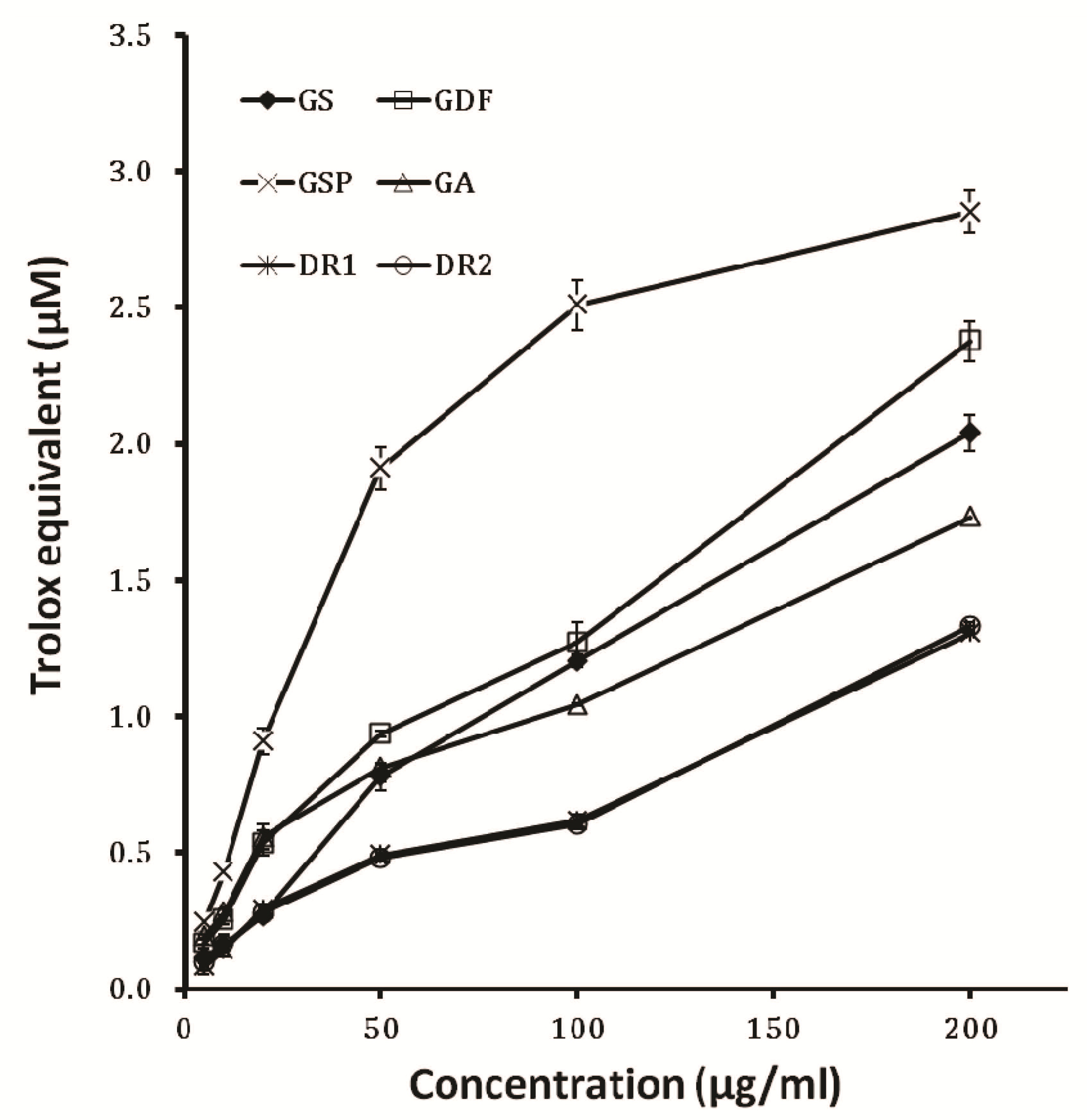

Supplement: Supplementary file 4 — Authors’ original file for figure 4 [file 40529_2013_46_MOESM4_ESM.jpeg]

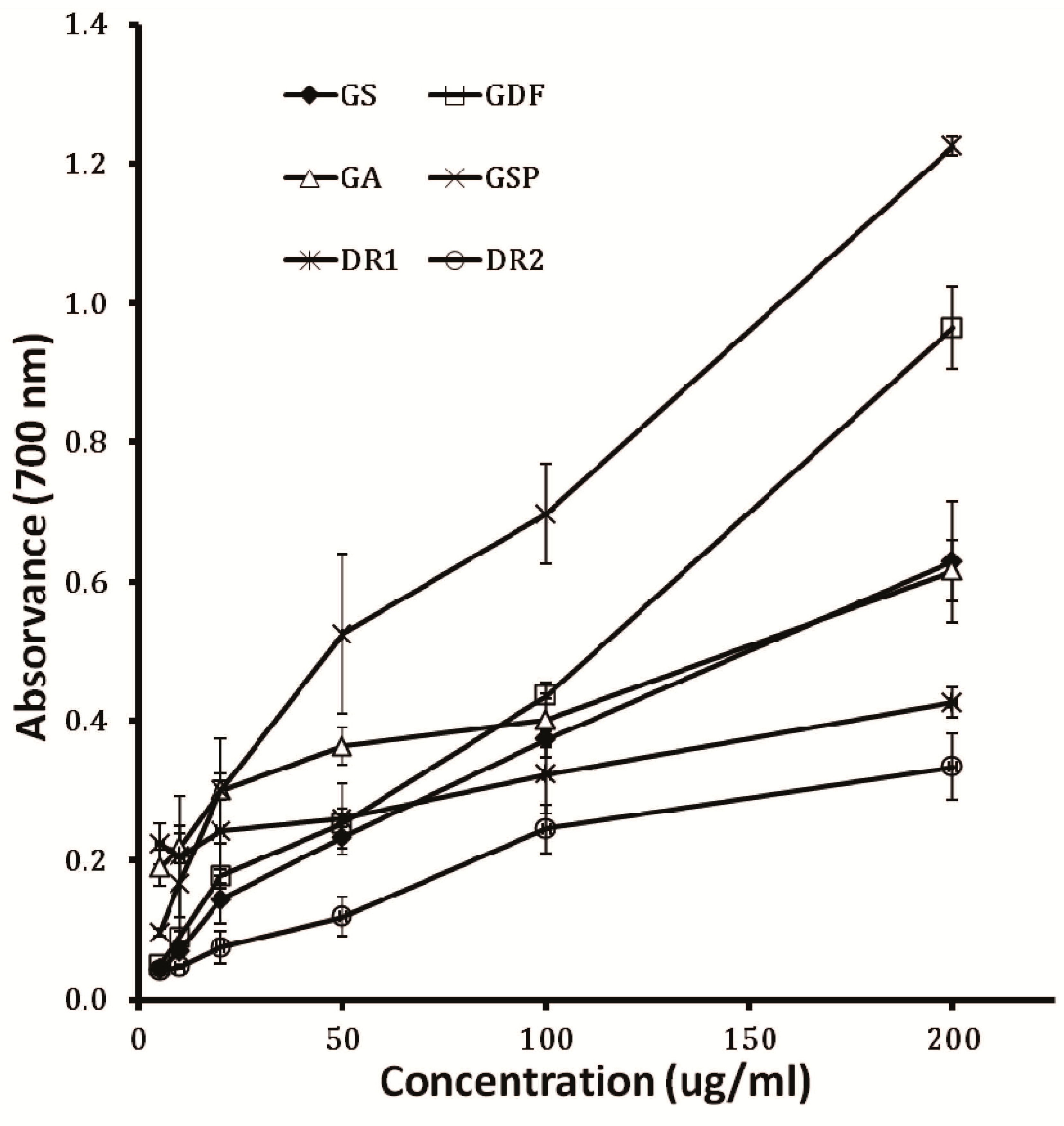

Supplement: Supplementary file 5 — Authors’ original file for figure 5 [file 40529_2013_46_MOESM5_ESM.jpeg]
